# Quantitative Assessment of Hippocampal Tau Pathology in AD and PART

**DOI:** 10.1007/s12031-020-01573-0

**Published:** 2020-05-05

**Authors:** Lei Zhang, Yankai Jiang, Jie Zhu, Huazheng Liang, Xiangyang He, Jiahong Qian, Hai Lin, Yubo Tao, Keqing Zhu

**Affiliations:** 1 China Brain Bank and Department of Neurology in Second Affiliated Hospital, Key Laboratory of Medical Neurobiology of Zhejiang Province, and Department of Neurobiology, Zhejiang University School of Medicine, 866 Yu Hang Tang Road, Hangzhou, 310058 China; 2 Department of Pathology, Zhejiang University School of Medicine, Hangzhou, 310058 China; 3 State Key Lab of CAD&CG, College of Computer Science and Technology, Zhejiang University, 866 Yu Hang Tang Road, Hangzhou, 310058 China; 4 Zhejiang University School of Medicine, Hangzhou, 310058 China; 5 Department of Neurology, Translational Research Institute of Brain and Brain-Like Intelligence, Shanghai Fourth People’s Hospital Affiliated to Tongji University School of Medicine, Shanghai, China

**Keywords:** Hippocampus, Tau pathology, Alzheimer’s disease, Primary age-related tauopathy

## Abstract

To quantitatively assess the distribution pattern of hippocampal tau pathology in Alzheimer’s disease (AD) and primary age-related tauopathy (PART), we investigated the distribution of phosphorylated tau protein (AT8) in 6 anatomically defined subregions of the hippocampal formation and developed a mathematical algorithm to compare the patterns of tau deposition in PART and AD. We demonstrated regional patterns of selective vulnerability as distinguishing features of PART and AD in functionally relevant structures of the hippocampus. In AD cases, tau pathology was high in both CA1 and subiculum, followed by CA2/3, entorhinal cortex (EC), CA4, and dentate gyrus (DG). In PART, the severity of tau pathology in CA1 and subiculum was high, followed by EC, CA2/3, CA4, and DG. There are significant differences between sector DG and CA1, DG and subiculum in both AD and PART.

Primary age-related tauopathy (PART) is defined recently by the presence of Alzheimer’s disease (AD)-type neurofibrillary changes without or with few Aβ plaques. The working classification for definite PART is based on the presence of neurofibrillary tangle (NFT) along with Braak stage ≤ IV and Thal Aβ phase as 0 (Crary et al. 2014). It remains a debate whether PART is a subtype of AD or a distinct tauopathy different from AD (Duyckaerts et al. 2015; Jellinger et al. 2015). How to differentiate PART from AD is an important issue to solve.

The hippocampus is an early region demonstrating tau pathology in both PART and AD. A recent study has indicated differences in the pattern of hippocampal tau pathology between classical AD and PART (Crary et al. 2014). Retrospective semiquantitative assessment of tau pathology in hippocampal subregions indicated more severe involvement in CA2 in PART cases than other regions in classical AD (Jellinger 2018). In view of these preliminary results and a number of divergent data, further quantitative assessment of the distribution pattern of hippocampal tau pathology in AD and PART is needed.

In the present study, we investigated the distribution of phosphorylated tau protein (AT8) in 6 anatomically defined subregions of the hippocampal formation and developed a mathematical algorithm to compare the patterns of tau deposition in PART and AD. Here, we demonstrated regional patterns of selective vulnerability as distinguishing features of PART and AD in functionally relevant structures of the hippocampus.

We examined 25 cases with AT8 positive tau pathology in the China Brain Bank (68% males, mean age at death 84.32 ± 9.46 years, 15 AD cases: Braak tau stages III–VI, Aβ Thal scores 1–5; and 10 PART cases: Braak stages II–IV, Aβ Thal scores 0). Neuropathological assessment included immunohistochemistry for Aβ, phospho-tau (antibody AT8). Tau pathology was quantitatively assessed in 6 major regions of the hippocampus, i.e., the entorhinal cortex (EC), CA1, CA2/3, CA4, subiculum, and the dentate gyrus (DG) using a computer quantification method. Statistical analysis was performed to compare AT8 immunoreactivity scores for NFT pathology in AD and PART cases using the Mann–Whitney *U* test. A significance level of 0.05 was used (Table 1).

**Table 1:** Mean values of neuronal tau pathology in various hippocampal areas in Alzheimer’s disease (AD) and primary age-related tauopathy (PART) relative to Braak neuritic stage

	Braak stage			
	III (*n* = 3)	IV (*n* = 9)	V (*n* = 1)	VI (*n* = 2)
AD				
Dentate nucleus	1.2	11.8	2.8	18.3
CA4	9.7	13.4	14.6	11.7
CA2/3	10.5	18.7	25.9	32.3
CA1	34.7	45.4	20.3	49.4
Subiculum	27.3	37.4	20.8	45.0
Entorhinal cortex	6.5	15.8	21.4	33.4
	Braak stage			
	II (*n* = 3)	III (*n* = 3)	IV (*n* = 4)	
PART				
Dentate nucleus	0.0	1.2	18.0	
CA4	2.1	4.1	11.2	
CA2/3	10.6	7.8	24.2	
CA1	7.6	13.0	33.9	
Subiculum	3.4	21.6	31.1	
Entorhinal cortex	8.2	13.2	27.8	

Since there are thousands of NFTs in each image of the hippocampus, it is labor-intensive and prone to error (e.g., missed NFTs) to identify all NFTs in the large image manually for human experts. Therefore, the present study employed a deep learning method for NFT detection to improve both efficiency and accuracy. Human experts first annotated some NFTs in the images, and then trained a YOLO model (Redmon et al. 2016), a neural network for real-time object detection, based on annotated NFTs as training examples. The trained YOLO model was used to detect NFTs in all images of the hippocampus of 25 cases, and human experts then confirmed whether NFTs detected by the YOLO model were true. Confirmed NFTs were used to update the YOLO model incrementally, the YOLO model was further used to detect NFTs in the images, and newly detected NFTs were confirmed by human experts to improve the accuracy and completeness of NFT detection. After three rounds, nearly all NFTs in the images were detected. The number of NFTs per square millimeter in each major region was calculated for each case. According to the organizational structures, three equal-sized circles were randomly selected in each area (CA4, CA2/3, CA1, SUB, ENT), and the whole DG area were selected from the hippocampus at the same time. After that, the number (*N*) of correct marks (NFTs) and the pixel area sizes (*S*) in all the circles and DG were showed automatically. The real length (l) and width (*w*) of the image were obtained from the Cellsens software, and the pixel length (*L*) and width (*W*) were from the image properties. The true area sizes (*s*) = (*l* × *w*) / (*L* × *W*) × *S*. The density of NFTs (/mm^2^) in each circle and DG region can be obtained through *N* / *s* (Fig. 1). For inter-group and between-group comparison of means coming from three circles in the areas and DG region, the Mann–Whitney *U* test was used.

**Fig. 1:**
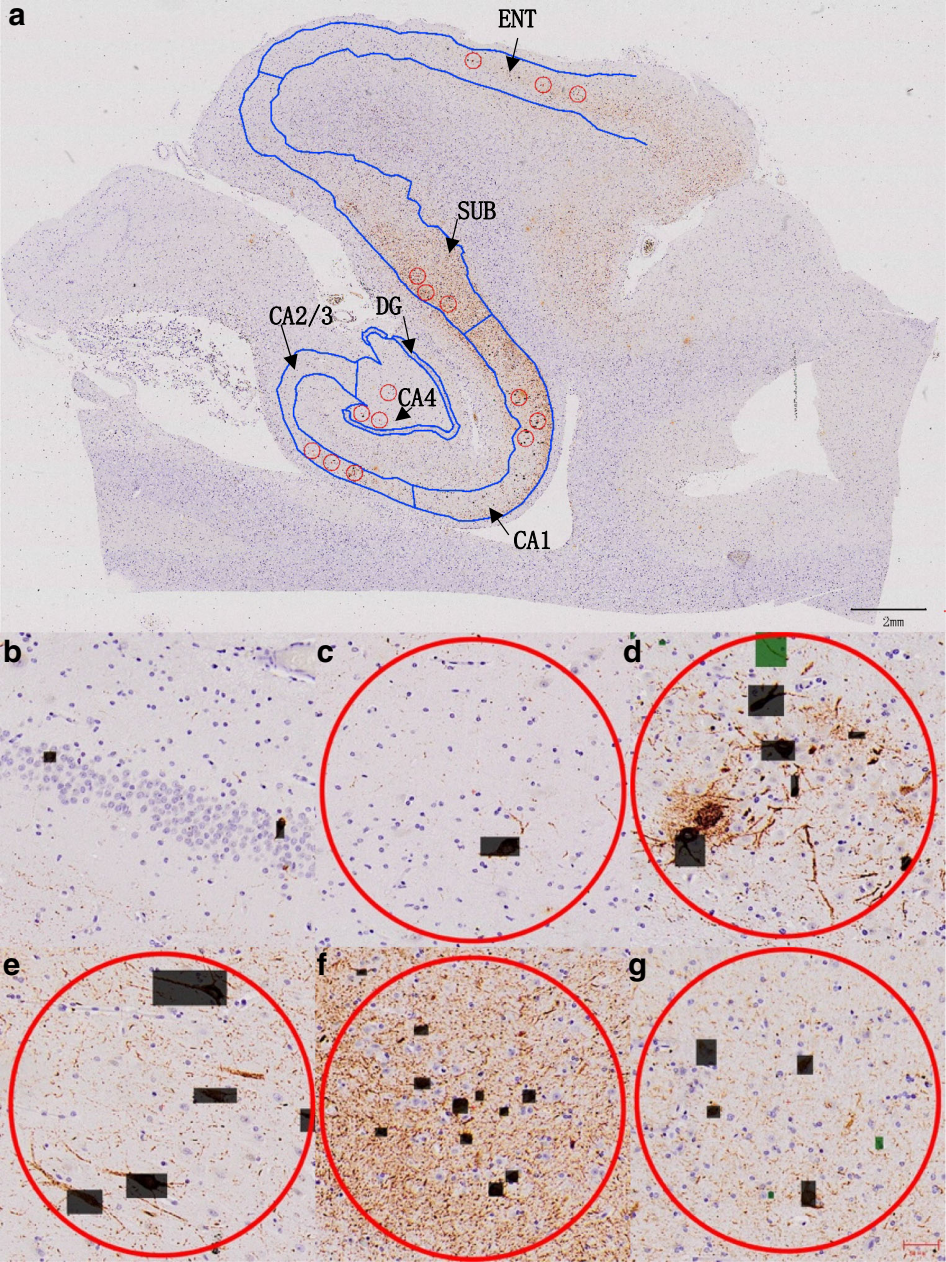
Immunohistochemistry of the medial hippocampal body in PART. (**a**) Detail of the medial hippocampal formation, three equal-sized circles were randomly selected in each area. (**b**) Granule cell layer of dentate gyrus (DG), (**c**) CA4, (**d**) CA2/3, (**e**) CA1, (**f**) subiculum (SUB), (**g**) entorhinal area (ENT). All boxes representing NFTs (neurofibrillary tangles) were automatically recognized by computer, the black ones indicated the marks corrected by manual judgment, and the green ones indicated the wrong marks. The red circles were randomly selected in each part (CA4, CA2/3, CA1, SUB, ENT), and every part had three circles. Scale bars (**a**) 2 mm, (**b**–**g**) 50 μm

PART cases showed increasing tau pathology in the EC (from 8.2 to 27.8/mm^2^), subiculum (from 3.4 to 31.1/mm^2^), and in CA1 (from 7.6 to 33.9/mm^2^) from Braak stage II to IV, while it was slightly higher in CA2/3 (from 10.6 to 24.2/mm^2^). Much less pathology was found in CA4 (2.1–11.2/mm^2^) and in particular, the DG (0–18/mm^2^). There was a statistical difference (*p* < 0.05) in tau pathology between DG and CA1, DG and subiculum, DG and EC in PART II, between DG and CA1, DG and subiculum in PART III, between CA4 and subiculum, CA4 and EC in PART IV. Statistical difference was also found between DG, subiculum, and EC when compared between Braak stage II and IV in PART (*p* < 0.05).

Among the AD brains of the present cohort, there is a limited number of stage V brains. A statistical difference in tau pathology was observed between DG and subiculum, DG and CA1, CA4 and CA1 at Braak stages IV (*p* < 0. 05). There was a significant difference (*p* < 0. 05) in the pattern of tau pathology in EC between AD and PART at Braak stages IV. Tau pathology in EC of PART (27.8/mm^2^) was more severe than that of AD (15.8/mm^2^) at Braak stages IV.

In a large Mayo AD series, CA2/3 were much less affected than CA1 by tau pathology (Murray et al. 2011). Some researchers did not observe significant differences between CA1 and CA2 in AD (Milenkovic et al. 2014); others reported milder involvement of CA2 at least in early or intermediate stages of AD (Walker et al. 2018). The severity of tau pathology in CA1 of AD cases of the present cohort was high, whereas it was much lower in DG. In Braak stages III and IV AD cases, tau pathology was high in both CA1 and subiculum, followed by CA2/3, EC, CA4, and DG. This pattern was similar to that of the PART cohort. In PART, the severity of tau pathology in CA1 and subiculum was high, followed by EC, CA2/3, CA4, and DG, whereas there were no essential differences in tau pathology between AD and PART at Braak stages III and IV, and PART cases (Braak stages III–IV) showed slightly more tau positive neurons in CA1 than CA2/3. In conclusion, the present study has shown quantitative tau pathology patterns of the hippocampus in AD and PART, which are similar to each other. There are significant differences in tau pathology between DG and CA1, DG and subiculum in both AD and PART.

## Data Availability

The datasets supporting the conclusion of this study are included in this article.
